# Identification of metacommunities in bioregions with historical habitat networks

**DOI:** 10.1002/ece3.70076

**Published:** 2024-08-08

**Authors:** Nivedita Varma Harisena, Adrienne Grêt‐Regamey, Maarten J. Van Strien

**Affiliations:** ^1^ Planning of Landscape and Urban Systems PLUS, Department of Civil Environmental and Geomatic Engineering ETH Zurich Zurich Switzerland

**Keywords:** biodiversity, conservation, habitat networks, landscape history, metacommunity

## Abstract

Although metacommunity theory provides many useful insights for conservation planning, the transfer of this knowledge to practice is hampered due to the difficulty of identifying metacommunities in bioregions. This study aims to identify the spatial extent of metacommunities at bioregional scales using current and historical habitat data, especially because contemporary biodiversity patterns may be a result of time‐lagged responses to historical habitat configurations. Further, this estimation of the metacommunity spatial extent is based on both the habitat structure and the dispersal ability of the species. Focusing on dragonfly and damselfly (odonate) species in the eastern Swiss Plateau, the research uses wetland habitat information spanning over 110 years to create a time series of nine habitat networks between 1899 and 2010. From these networks, we identified the spatial extents of metacommunities based on the year of habitat information as well as on watershed boundaries. To identify the best metacommunity spatial extents, the study investigates whether patch pairs within a metacommunity exhibit greater similarity in species composition (i.e. lower beta‐diversity) than patch pairs between metacommunities. For the different metacommunities, we further investigated correlations between gamma diversity and metacommunity size and compare them to theoretical expectations. In both analyses we found that augmenting spatial metacommunity identification with historical geographical proximity results in stronger associations with biodiversity patterns (beta and gamma diversity) than when using only current‐day habitat or watershed information.

## Graphical abstract

This study employs current and historical habitat data to spatially identify and classify metacommunities in the eastern Swiss Plateau, focusing on dragonfly and damselfly species. Analysing wetland habitat information spanning 1899 to 2010, the research finds that augmenting spatial metacommunity identification with historical geographical proximity enhances associations with biodiversity patterns, resulting in stronger correlations for beta and gamma diversity compared to using only current‐day habitat or watershed information.

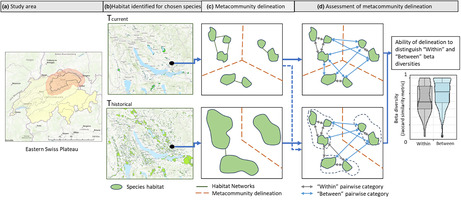

## 1 INTRODUCTION

Biodiversity in a constantly changing landscape is a complex subject to understand, especially when trying to uncover the underlying processes by which the change influences species diversity (Tscharntke et al., 2012). The assembly of ecological communities, as most other natural phenomena, show traits of self‐organisation, that is bottom‐up interactions at local scales leading to emergent regional community dynamics (Leibold & Norberg, 2004; O'Sullivan et al., 2019; Serván et al., 2018). Such multiscale patterns of community assembly and development have been researched under the umbrella theory of metacommunities (Hubbell, 2001; Leibold et al., 2004; Leibold & Miller, 2004; Logue et al., 2011). A metacommunity is composed of a network of linked communities of species that assemble through interrelated processes, such as dispersal limitations, biotic interactions, and environmental changes (Brown et al., 2017; Mouquet & Loreau, 2003; Thompson et al., 2020; Winegardner et al., 2012).

Different frameworks have been theorised and empirically identified to understand the assembly of metacommunities (Brown et al., 2017; Leibold et al., 2004; Thompson et al., 2020; Winegardner et al., 2012). The ‘neutral’ assembly framework elucidates how assembly is driven by spatial mechanisms, such as dispersal and/or by stochastic processes (Economo & Keitt, 2008, 2010; Hubbell, 2001), whereas in the ‘niche‐based’ assembly framework, habitat heterogeneity and species‐specific characteristics, such as ecological niche, dispersal ability and competition traits, additionally can define metacommunity dynamics. Such process‐based frameworks of metacommunity assembly can provide information to better understand regional diversity in relation to landscape connectivity, which can further promote species diversity in landscapes (Fournier et al., 2017; Thompson et al., 2014).

Not only contemporary, but also the history of above‐mentioned processes within the backdrop of a changing landscape, can be important to explain current community composition, especially in landscapes where anthropogenic impacts have rendered the habitats smaller and fragmented over time (Ewers et al., 2013). It has been empirically proven that in small and fragmented habitats, populations of species tend to decline faster when compared to larger, well‐connected habitats (Chase et al., 2020; Hanski & Ovaskainen, 2000; Horváth et al., 2019; Rybicki & Hanski, 2013). Yet the species response to such past connectivity changes can be lagged, with previously connected habitats continuing to share a common species pool, indicating a path dependence on past habitat change (Bennett & Saunders, 2010; Chase, 2003; Jamin et al., 2020; Jung et al., 2020; Tappeiner et al., 2020). This delayed response in species diversity is called an extinction debt, which can be defined as possible current or future species extinctions or population declines that can be attributed to historical landscape changes (Kuussaari et al., 2009; Lira et al., 2019; Watts et al., 2020). Thus, metacommunity compositions must be considered a result of mechanisms interacting in varying scales in both spatial and temporal dimensions (Guzman et al., 2022; Rapacciuolo & Blois, 2019).

At bioregional scales, multiple metacommunities may exist that are separated by a lack of dispersal, and therefore, it is important to estimate the extents of different metacommunities. Such spatial identifications enable the translation of information from metacommunity theory into practical on‐the‐ground conservation strategies. Many metacommunity studies are spatially implicit and formulate species movement as space‐independent dispersal rates (Dong et al., 2021; Etienne et al., 2019; Leibold et al., 2004; Suzuki & Economo, 2021; Valanko et al., 2015). Traditionally, in the few spatially explicit studies, metacommunities are identified based on geographically proximate sites of habitats (Leibold & Chase, 2018). For example, Maurer et al. (2013) aimed to identify the best metacommunity geographical extent for a focal community based on relative abundance, phylogenetic and environmental similarity indicators for certain species. However, the delineation of metacommunities at a bioregional scale empirically has been quite subjective and is usually user‐defined (Leibold & Chase, 2018), leading to highly context dependant analyses. There is thus a need to develop methods to identify metacommunities spatially in real landscapes.

For species communities in patchy habitats, habitat networks are a useful way to identify regional clusters of patches (i.e. metacommunities) based on the spatial distance by which they are separated. For a certain selection of species, habitat networks allow for metacommunities within a bioregion to be identified using bottom‐up local interactions. The local interactions are based on the dispersal limits of the selected species (Economo, 2011; Suzuki & Economo, 2021). Network attributes, additionally, can indicate how ‘connected’ or ‘isolated’ patches are and can have implications on species assembly based on how accessible nearby patches are for colonisation (Borthagaray et al., 2015; Economo & Keitt, 2010). Networks are also useful in tracking connectivity change over time. Topological metrics derived for networks defined for different time steps can represent the impact of the sequence of habitat loss and patch configuration changes on metacommunity composition (Thompson et al., 2017). Integrating such information to create time series of historical network models can help track metacommunity dynamics as a response to changing landscapes over time.

There are several diversity indices that can be used to analyse mechanisms of species assembly in metacommunities. One such index is beta‐diversity, which is commonly defined as the similarity in composition of species between habitat patches (Anderson et al., 2011; Baselga, 2010). Beta‐diversity provides a link between local and regional species diversity (Chiantore et al., 2018; Svenning et al., 2011; Tuomisto, 2010; Viana et al., 2016) and is a useful indicator in rapidly changing environments as it can provide an estimate of how ‘far’ ecosystems have drifted apart (Enkhtur et al., 2021; Strengbom & Cugunovs, 2020). Since dispersal limitation is an important mechanism for the assembly of metacommunities and a distance decay in species composition similarity has been well established (Leibold & Chase, 2018; Nekola & White, 1999; Soininen et al., 2007), beta‐diversity can be a useful metric to estimate metacommunity extents. Another useful index to quantify the species composition in metacommunities is gamma diversity, which is the total number of species in a metacommunity at a regional scale. Gamma diversity has been hypothesised to linearly scale with metacommunity size, which is quantified based on geometric properties of regional metacommunities (Economo & Keitt, 2008). Therefore, identified metacommunity geographical extents can be used to estimate these relationships and check whether the expected theoretical relationships are observed.

In this study, we use current and historical habitat data to estimate the spatial extent of metacommunities at bioregional scales. We hypothesise that metacommunity spatial extents identified using historical geographical proximity of habitats better correlate with contemporary biodiversity (beta and gamma diversity) patterns than current habitat configurations. We test this hypothesis for dragonflies and damselflies (i.e. odonate) species that have been monitored in wetland habitats in the eastern Swiss Plateau region using spatial habitat networks traced over 110 years (1899–2010). Making use of time series of habitat networks, we identify the most likely metacommunities by checking the within‐ and between‐metacommunity beta‐diversity, as well as by investigating gamma‐metacommunity size relationships. We also test the identification of metacommunities based on abiotic information in the form of watershed delineations. In some studies, the extent of metacommunities is expressed as a certain distance from a central habitat patch or community (e.g. Maurer et al., 2013). For each central patch, this approach essentially leads to a different selection of patches being grouped into a metacommunity. In contrast to this approach, our approach aims to identify the extent of spatially discrete metacommunities at a regional scale for a certain taxonomic group.

## 2 MATERIALS AND METHODS

**FIGURE 1:**
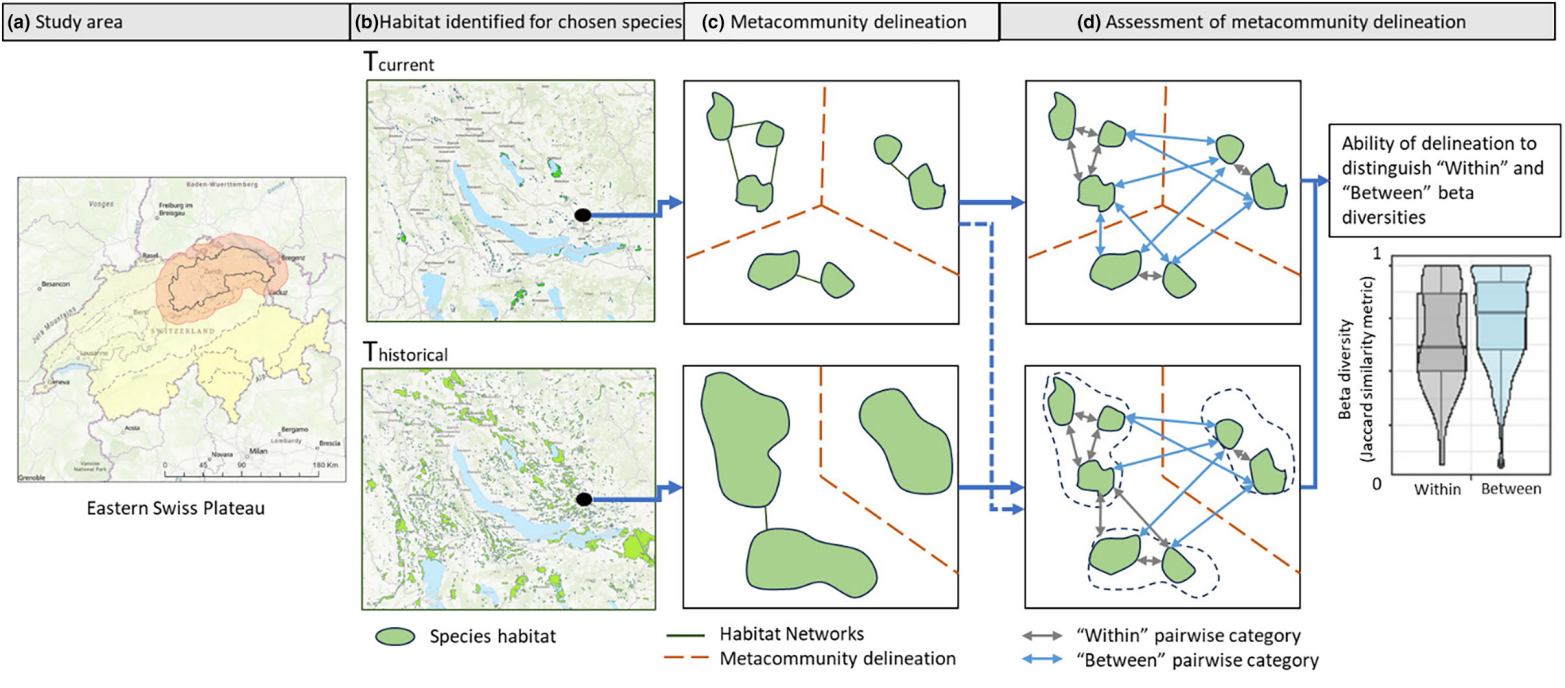
(a) Map of the study area, that is the Eastern Swiss Plateau region (black line) with a 15 km buffer (shaded red) indicating the study area; (b) A zoomed in map of the habitat patches for the region around the city of Zurich both for the current time step (2010) and an historical time step (1899); (c) Schematic overview of how the metacommunity spatial extents are delineated based on connected patched that fall within a species dispersal threshold; (d) Schematic overview patch pairs sorted into ‘within’ and ‘between’ categories based on the metacommunity spatial extents along with exemplary boxplots used to assess the best delineation.

Figure 1 shows the workflow of this study to identify odonate metacommunities in the eastern Swiss Plateau bioregion with the help of spatial habitat network models. The basic workflow includes the estimation of metacommunity spatial extents based on both the species dispersal limitations and the habitat structure within the landscape. This is done for both the current time step (2010) and for historical time steps (1899–1992). We use spatial habitat networks for multiple dispersal distances to estimate connected groups of patches that we identify as metacommunities. These metacommunity extent estimations are then related to odonate species diversity similarities within and between the metacommunity extents to assess the delineation that is most representative for the odonate communities in the study region.

### 2.1 Study area and historical data

Wetlands are essential habitats that, at the interface of terrestrial and aquatic, form ideal habitats for a wide range of species (CBD, 2015; Fluet‐Chouinard et al., 2023). Wetlands are typically also identified as ‘patchy’ landscape elements, as they are usually surrounded by more elevated land that tends to isolate them (Bertassello et al., 2021; Leibowitz, 2003). Furthermore, connectivity amongst such patches has been found to be very important for the species that inhabit these areas, such as dragonflies and amphibians (Watts et al., 2015; Zamberletti et al., 2018). Smaller patches of wetlands, which are usually overlooked in protection legislations, have also been found to contribute important structural properties to the wetland network (Gibbs, 1993; Semlitsch & Bodie, 1998; Watts et al., 2015).

The study area for this research includes all the wetland patches in the eastern Swiss Plateau (Figure 1a), which is a distinctive biogeographical region (BAFU, 2022). The Swiss plateau is one of the most densely populated regions in Europe and encompasses many wetlands that have strongly declined in number and size in the past century (Delia Vega Orozco et al., 2015; Gimmi et al., 2011; Kienast, 1993; See Appendix A). The eastern Swiss Plateau has additionally shown a larger proportion of wetland loss over time (Müller et al., 2018). We buffered this region with 15 km to minimise edge effects. Our final study area was approx. 4474 km^2^ (Figure 1a).

Information on wetland patches for the different time steps was segmented from several historical topographic map series: Siegfried maps (1870–1926; Wu et al., 2022), old national maps (1938–1994; Wu et al., 2023). The 2010 wetland data was extracted from the vectorised topographic map ‘Swiss Map Vector 25’ (Swisstopo, 2021). Multiple historical map sheets that include information on the eastern bioregions in the Swiss Plateau were combined for 8 times steps which are: 1899, 1918, 1933, 1949, 1959, 1970, 1978 and 1992. These time steps were chosen so that full coverage of the Swiss Plateau was obtained with sheets that were not used in other time steps (Räth et al., 2023). We found that some wetlands in the different time steps did not perfectly overlap, due to errors in digitisation and due to the different data sources for the different time steps. These were corrected by adding all wetland pixels identified in the later time steps to its earlier counterparts using raster calculation, that is if a pixel in a time step is identified as wetland with no ancestor, we replicate it as wetland in all the previous time steps. This modification was based on the realistic assumption that no new wetlands emerged over time in this region of the Swiss Plateau (i.e. the majority of historical wetlands have disappeared or reduced in size; FOEN, 2017; Müller et al., 2018).

### 2.2 Beta and Gamma diversity of Odonata species

Dragonfly and damselfly (odonate) species have been identified as key indicator species for freshwater wetland ecosystems and other aquatic systems, because of their sensitivity to human disturbance and land use change (Kutcher & Bried, 2014; Monteiro et al., 2015; Rocha‐Ortega et al., 2019). Damselfly species show a time‐lagged response to landscape change (Rocha‐Ortega et al., 2019). In Switzerland, communities of odonates have faced changes in composition over the past 50 years, but few species have gone completely extinct (Gonseth & Monnerat, 2003). The species occurrence data used in the study were taken from the red list monitoring programme that sampled multiple sites throughout Switzerland during the years 2010 to 2015 (Gonseth & Monnerat, 2002). According to this dataset, there are currently 75 species occurring in the Swiss Plateau of which 11 are critically endangered. As monitoring studies aim to assess species diversity and visit sites repeatedly, we assumed the dataset to be a reliable estimate of the species composition at each site. We only included those species observations that were coinciding geographically with areas characterised as wetlands in 2010. The final data included species composition in 88 wetland patches across the eastern Swiss Plateau. For all these patches, we calculated the inter‐patch beta‐diversity of odonates using the Jaccard dissimilarity metric. This metric has been widely used to estimate beta‐diversity when occurrence, but no abundance, data of species is available (Lu, 2021). Given that the distance decay in similarity of species communities across geographic distances have been proven repeatedly using beta‐diversity (Leibold & Chase, 2018; Nekola & White, 1999; Soininen et al., 2007), the Jaccard similarity metric can provide useful information for metacommunity delineation in the absence of abundance‐based datasets. We further calculated the gamma diversity by calculating the total species richness in a delineated metacommunity region (see Section 2.6 for more detail).

### 2.3 Construction of current and historical spatial habitat networks

We created habitat networks in which nodes represented wetland habitat patches and edges represented the geographical proximity of patches (see Figure 1b), which we calculated using a Euclidean distance threshold with the graph4lg R‐package (Foltête et al., 2021; Savary et al., 2021). Although methods exist based on resistance surfaces that also consider the permeability of the landscape between habitats to quantify connectivity (Albert et al., 2017; Meyer & Larroque, 2022), we only considered Euclidean distance as historical data to parameterise past landscape resistance surfaces was missing. Nevertheless, we calculated the ‘network distances’ between pairs of habitat patches making use of a least‐cost path algorithm. As we used a homogeneous resistance surface, the calculated distances are effectively Euclidean. However, this methodological approach gave us the flexibility to test alternative resistance surfaces in future research.

We tested five different dispersal thresholds, 250, 500, 750, 1000 and 2000 m, to create the links between the habitat patches. These distances were selected as they are representative of dispersal distances found in odonates (Angelibert & Giani, 2003; Conrad et al., 1999). Distances between patches are computed as edge‐to‐edge straight‐line geographic distances and are assigned as edge weights. Thus, given a certain dispersal threshold, patches are connected if their edge‐to‐edge distance is below this threshold or if there are steps of intermediate patches (so‐called stepping stones) whose inter‐patch distances each are below the threshold (Economo & Keitt, 2010; Saura et al., 2014). If a patch has no neighbouring patch within a given dispersal threshold it will not have any connected edge assigned to it and is thus an isolated patch.

We constructed habitat networks for each of the nine time steps of which the ones from 1899–1992 (eight time steps) are considered historical networks and the one of 2010 is considered the current habitat network, since it overlaps with the time period of species data collection. The 2010 habitat network represents the status quo, as most studies on habitat connectivity only use current habitat networks (e.g. Ortiz‐Rodríguez et al., 2019). This network is thus an important reference network to compare the historical networks with.

### 2.4 Metacommunity delineation

The delineation of metacommunities in this study is undertaken by identifying network components within the larger network for each year in the study area. Network components are defined as groups of connected habitat patches with no links outside of their own set of nodes. It can be assumed that due to the higher geographical proximity of the patches within a component than between components, most species interactions would occur within a component and thus can be identified as metacommunities of species. Components (or metacommunities) were identified in both the current and the historical networks. As habitat patches in the past were more numerous, larger in area and, therefore, better connected than contemporary patches, network components were larger in the earlier time steps. This analysis was repeated for the different dispersal thresholds.

The network components or metacommunities are visualised using polygons. These metacommunity extent estimations serve as a grouping polygon based on which patch pairs were either identified as ‘within’ a metacommunity or ‘between’ metacommunities (Figure 1c). Their geometry per se does not provide any information, as the polygon boundaries were based on defining a partition between clusters of geographically closer patches and the true metacommunity boundaries can be fuzzy. Additionally, because of the presence of isolated patches (i.e. patches without any connections), some single patch metacommunities are also identified; however, these are not used for assessment.

In addition to the network‐based metacommunity delineations, we also tested whether metacommunities could be identified with geographical abiotic information. For this, we used, watersheds (termed ‘WS’) as defined by the Swiss Federal Office for the Environment based on 150 m contour lines (BAFU, 2006).

### 2.5 Assessing the best metacommunity delineation

Metacommunity delineations are first assessed based on their ability to differentiate current patch‐pair beta‐diversity similarities ‘within’ and ‘between’ metacommunities (Figure 1d). In other words, we compared the beta‐diversity of current patch pairs ‘within’ a delineated metacommunity to that of patch pairs ‘between’ metacommunities in a 15 km surrounding (see Figure 1 for graphical representation of this). This 15 km distance limit was chosen, as the inclusion of patch pairs beyond this distance created a dissimilarity saturation (i.e. an excess of 1 values for beta‐diversity; Brown et al., 2017), which hampered proper analysis of the data. This analysis is based on the assumption that beta diversities are on average lower for patch pairs falling ‘within’ a delineated metacommunity, than for patch pairs where each patch belongs to a different metacommunity (Chase, 2003; Economo & Keitt, 2010). The identified metacommunities at each time step are used to group the current patches differently (i.e. grouped as either ‘within’ or ‘between’; as can be seen in Figure 1c). This leads to a re‐distribution of the within and between patch pairs, but not a reduction in total number of patch pairs. Thus, the final aim is to relatively assess which spatial metacommunity configuration results in the largest difference in distributions of beta diversities ‘within’ than ‘between’ metacommunities and in the right direction, that is mean ‘within’ diversity is lower than the mean ‘between’ diversity. We used a two‐sided t‐test to check whether the distributions of the ‘within’ and ‘between’ beta diversities were significantly different.

We further used classification accuracy metrics and the Kappa coefficient (DeVellis, 2005; Kulkarni et al., 2020) to identify how accurately the ‘within’ and ‘between’ patch‐pair categories differentiated the beta‐diversity values. As we had a binary categorisation of patch pairs into ‘within’ and ‘between’ metacommunity classes, we also binarised the pairwise beta‐diversity measures into ‘high’ and ‘low’ using the median beta‐diversity (Jaccard index ≈ 0.8) as classification threshold (i.e. half of the beta‐diversity values were below the threshold, whereas the other half was above this threshold). Thus, accuracy indicates the ability of metacommunities to group patch pairs and beta‐diversity classes into the expected categories (‘within’ patch pairs correspond to ‘low’ beta‐diversity; ‘between’ patch pairs correspond to ‘high’ beta‐diversity) and the Kappa value additionally provides a score of agreement correcting for a random chance of agreement between the categories. Therefore, the higher the Kappa score the better the performance in correctly differentiating beta‐diversity.

### 2.6 Metacommunity based network metrics and correlation to regional diversity

Metacommunity or regional species diversity (i.e. gamma diversity) is very useful in estimating larger scale biogeographical and assembly processes that cannot be captured at local (habitat) level species diversity. As per Economo and Keitt (2008), metacommunity size, defined as either area or mean shortest distance between all patches falling within a metacommunity (i.e. network diameter), is expected to correlate positively with gamma diversity. This is analogous to the species‐area relationships identified in other macroecological studies that also fall within the purview of gamma diversity (Leibold & Chase, 2018). We use this information as an additional test to estimate the species‐area (and diameter) relationship at regional scale based on historical and current definitions of metacommunities. Additionally, we also test the no. of patches in a metacommunity and gamma diversity relationship similarly.

## 3 RESULTS

### 3.1 The delineation of metacommunities with temporal information

**FIGURE 2:**
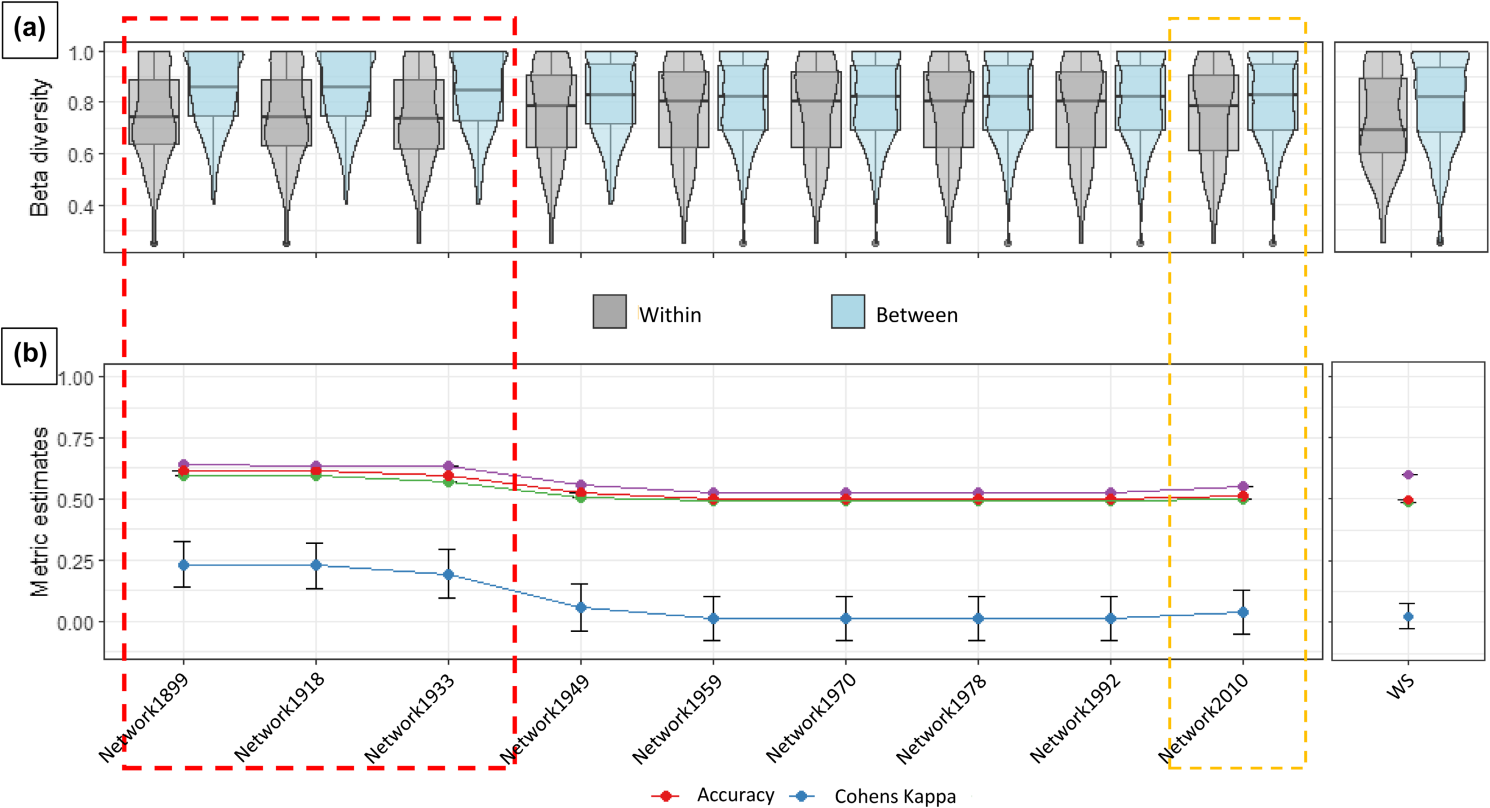
Results showing classification accuracy of different historical (Networkxxxx, where xxxx stands for the year), current (Network2010) and watershed (WS) based metacommunity clusters for within‐ and between‐metacommunity beta‐diversity at 500 m dispersal threshold (a) boxplots of the ‘Within’ and ‘Between’ categories of patch pairs and their corresponding beta‐diversity distributions; (b) Kappa scores, Accuracy and Precision metrics for the classification; Red dotted box shows networks with relatively high Kappa values and yellow dotted box shows the 2010 (current) network.

We found that the metacommunities derived from the historical networks from 1899 to 1933 (red box in Figure 2) more accurately classified low and high beta‐diversity than the current (2010) network (yellow box in Figure 2). For the most representative metacommunity structure, we expected that the ‘within’ metacommunity beta diversities were on average lower than the ‘between’ metacommunity beta diversities. This expectation was confirmed for metacommunities defined from networks of 1899, 1918 and 1933. For these networks, we found relatively high Kappa values (red dashed box in Figure 2b) and lower median beta‐diversity values for the patch pairs ‘within’ (grey boxplots in Figure 2a) when compared to the patch pairs ‘between’ (blue boxplots in Figure 2). The differences between the distribution of beta diversities within and between metacommunities were significant (*p* < .01) for the metacommunity definitions from 1899, 1918 and 1933. This also holds for the current network (See Appendix C for details), implying that the current network also significantly identifies ‘within’ patch pairs as having lower mean beta‐diversity than the ‘between’ patch pairs (yellow box in Figure 2a), however, the frequency of mismatched cases result in a Kappa score of approx. 0 (yellow box in Figure 2b). This indicates that the within‐ and between‐metacommunity patch pairs derived from the current network are not capable of differentiating between high and low beta‐diversity any better than by random chance. The same result is seen for metacommunities identified by the watersheds. It is noteworthy that the watershed delineation encompasses many patch pairs with low beta‐diversity within its boundaries (grey bar in the ‘WS’ boxplot in Figure 2a); however, there are also many low beta‐diversity patch pairs between the boundaries of wetlands that result in a low Kappa value (‘WS’ in Figure 2b). In summary, the metacommunity delineation derived from habitat networks from 1933 and before can be considered most representative for odonate species within the wetlands of eastern Swiss Plateau.

These results were evident when habitat networks were constructed by considering inter‐patch dispersal distances of 500 and 750 m, whereas lower and higher dispersal distances do not show similar patterns (See Appendix B). Based on the overall highest Kappa values, we regarded the components in the 1899 habitat network as most representative for the true metacommunity structure of odonates in the eastern Swiss Plateau and used these metacommunities in the further analysis.

### 3.2 Mapping network‐based metacommunities

**FIGURE 3:**
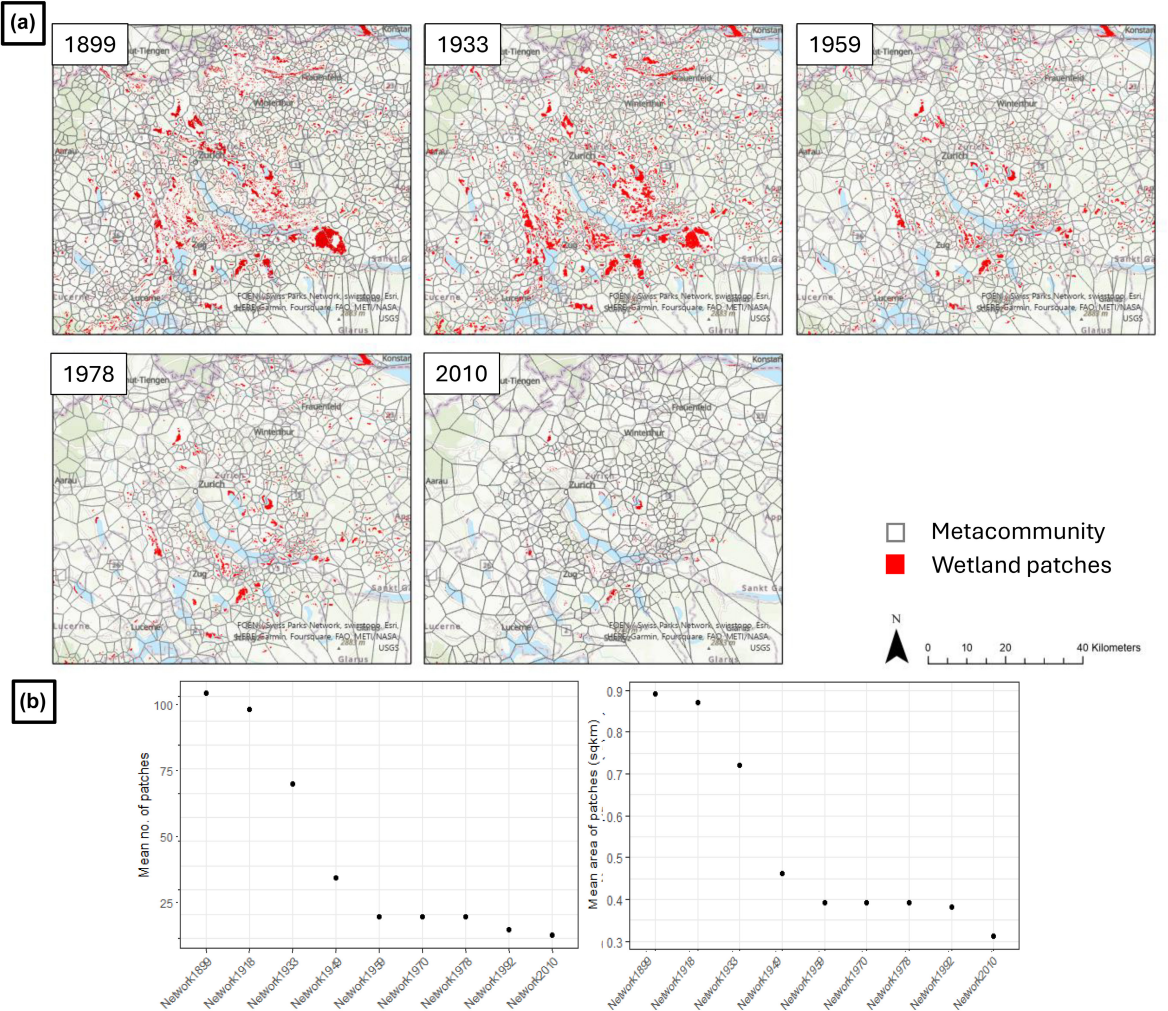
(a) Decrease in wetland cover from 1899 until 2010 with metacommunity boundaries (black polygons); (b) Change in mean no. of patches and mean area of patches (based on 500 m maximum dispersal) over 1899–2010 for all metacommunities with odonate species data available.

The delineation of the metacommunities based on dispersal limits is exemplified for five‐time steps between 1899 and 2010 (Figure 3a). The maps show a clear decrease in the number of wetland patches over the years. Figure 3b shows the decrease in average number of patches per metacommunity and mean area of patches over the years as the wetland patches have become smaller or completely disappeared. It can be seen from the graphs that until 1949 there was a stark reduction in number and size of wetlands. Post 1949 there is a stagnation of these trends, with both metrics showing lower variability until 2010. This is in line with previous research that shows that especially between 1900–1950 there were strong reductions in wetlands in the Swiss Plateau (Gimmi et al., 2011; Müller et al., 2018). It is to be noted that the statistics denote only those metacommunities that have species information in it in more than one patch, since otherwise the ‘within’ metacommunity beta‐diversity cannot be computed.

### 3.3 Regional diversity in metacommunities

**FIGURE 4:**
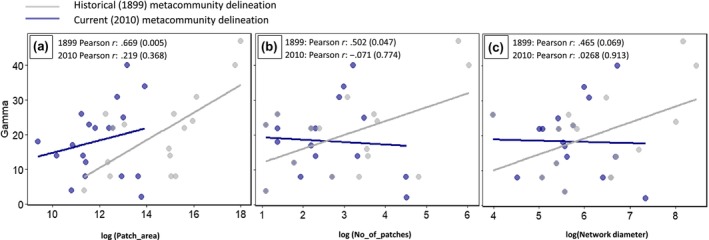
Scatter plots showing the relationship between log transformed (a) patch area, (b) no. of patches and (c) network diameter of metacommunities with corresponding regional (gamma) diversity for both historical (1899) and current (2010) metacommunities. Pearson correlation values and corresponding *p*‐values (in brackets) are shown in the scatterplots.

Extending the analysis to regional diversity for different metacommunities in the Swiss plateau, the linear relationship between gamma diversity and metacommunity size (in terms of area and number of patches) is significant (*p* < .05) and positive, as expected from literature (Economo, 2011; Economo & Keitt, 2008), only when using metacommunities derived from the habitat network configuration in 1899 (Figure 4a,b). The mean shortest distance or the network diameter estimated from the 1899 metacommunity definition also shows a positive correlation to gamma diversity but is only weakly significant (*p* < .1; Figure 4c). This result along with the lack of significance for the diversity‐size relationship for metacommunities derived from the most current year (*p* > .05) shows that metacommunity delineations based on historical information tend to align better with theoretical regional diversity patterns (Brown et al., 2017; Logue et al., 2011).

## 4 DISCUSSION

### 4.1 Identifying metacommunity spatial extents

The study outlines a generic method to delineate metacommunities in a bioregion making use of historical habitat data. Traditionally most metacommunity studies are done at smaller scales, with spatial definitions being either non‐existent (non‐spatial), context‐specific or arbitrarily user‐defined (Leibold & Chase, 2018). Most network model based metacommunity studies do not explicitly investigate metacommunity geographic extents (Borthagaray et al., 2015; Economo & Keitt, 2008). Spatially explicit estimation of geographically separate metacommunities could be a bridge between metacommunity dynamics and regional conservation planning efforts (Savary et al., 2024). One of the few studies that looked into the identification of metacommunity sizes is Maurer et al. (2013), who aimed to identify metacommunity geographic extents based on similarity indices between focal communities and nearby locations. However, due to the inherent symmetry of Euclidean distance buffers, any anisotropic or directional influences of landscape structure is not captured in their approach. As can be seen from our results the metacommunities identified differ in geometry. This is because (i) network models inherently account for the anisotropic structure of the patches to define a connected module; and (ii) network models account for patch connectivity via intermediate patches (i.e. via stepping‐stones) allowing for larger metacommunity spans that can be more representative of species metacommunities. However, our study only considers landscape structure due to increasing geographical distance, and movement barriers such as roads with heavy traffic or fencing can be additional factors leading to anisotropic metacommunity structure. Including the permeability of the landscape matrix to define the network links can additionally uncover the importance of the landscape matrix in metacommunity dynamics (Albert et al., 2017).

Metacommunity spatial extent estimation in our study depends both on the dispersal abilities of the species group and the structure of their habitats in the landscape. It has been shown that landscape perception for a certain taxa based on its dispersal abilities can affect metacommunity structure (Borthagaray et al., 2015). It is important to note here that intraspecific differences within a taxon are not accounted for in our study, since we finally aim to identify a single metacommunity geographical extent delineation for a complete taxonomic group. Such intraspecific variations in traits within a taxonomic group can have additional effects on community structure (Violle et al., 2012). However, the presented methodology of identifying proximate groups of habitats that belong to a metacommunity is generalisable. Sensitivity tests with multiple dispersal distances can identify spatial clusters of habitats that strike the best balance between low beta diversities within the clusters and high diversities between clusters for a single taxon. This methodology could be useful in regional conservation planning, where metacommunities of different key taxonomic groups can be independently assessed for differentiated conservation efforts.

The identification of metacommunities in our study is based on the assumption that habitat patches that form one metacommunity will show on average higher similarities in species composition than communities that are in different metacommunities, and thus can be utilised to delineate metacommunity geographical extents. These extent definitions are not sharp as can be seen from the boxplots where there are overlaps in beta‐diversity values between the ‘within’ and ‘between’ categories and also in Kappa values that indicate low to moderate agreement (McHugh, 2012). Empirical metacommunity research is a complex undertaking and results can be confounded by uncertainties in species responses, traits and interactions along with potential biases in the empirical species observation data and (historical) habitat data (Logue et al., 2011). The overall high beta‐diversity in the Swiss Plateau (median Jaccard index = 0.8) could indicate that most odonate communities are quite isolated, which could be another factor that hinders a clearer identification of metacommunities. This can also indicate a lack of information in species composition‐based similarity that could be enhanced using abundance datasets. Additionally, we acknowledge that in ecological communities there are many variables that can influence biodiversity patterns, including those of patch or landscape quality (e.g. historic and current habitat management, variations in water levels) and biotic traits and interactions (e.g. species competition, niche widths). Variations in these variables over space and time can change the course of community assembly (Kuussaari et al., 2009; Paltto et al., 2006) and could thus have contributed to the ‘fuzzyness’ of the metacommunity spatial identification.

### 4.2 Importance of historical connectivity

Our analysis furthermore showed that odonate metacommunities defined using patch pairs falling within historically delineated boundaries were more similar in species compositions than pairs that were crossing these boundaries. This result could not be obtained with current habitat networks (at any of the dispersal distances; see Appendix B), which is evidence for the need to include historical geographical proximities to identify metacommunities. Metacommunities identified with watershed boundaries also were not able to differentiate well between low and high beta‐diversity, suggesting that mechanisms based on current abiotic environmental factors might not explain enough of the assembly of metacommunities. Thus, for regions that have undergone considerable losses in size and number of habitats, this study provides support for the need to conserve habitat connectivity at spatial scales that also encompass historical proximities.

As an additional support for metacommunity delineation, we found that metacommunities identified using historical habitat networks produced species diversity‐area relationships as expected from metacommunity theory (Economo & Keitt, 2008). The significant relationship between metacommunity size (area and number of patches) with gamma diversity estimated from historical networks, and the lack thereof for the current‐day habitat networks, points towards a possible broader regional structure in community assembly than can be defined using only current geographical proximity. We also found a weakly significant positive correlation between gamma diversity and network diameter, which was again not found for the current habitat networks. A lower network diameter points towards a more compact network structure, which can imply a higher efficiency in dispersal‐based assembly processes, thus a lower gamma diversity due to competition trade‐offs (Suzuki & Economo, 2021). However, these mechanisms are confounded due to the covariance of metacommunity area with network diameter, and it is thus difficult to solely identify the influence of compactness of network structure. A wider range of network metrics and their change over time could be useful to identify more topology‐based emergent attributes that can relate to community assembly patterns (Reunanen et al., 2012; Saura & Rubio, 2010; Savary et al., 2021). Future research incorporating such network metrics could increase the strength of the found relationships.

We found an influence of habitat network from more than 70 years ago on current diversity patterns. However, the exact mechanism by which the past influences the present still needs to be investigated. Historical networks represent the habitat configuration that includes common ancestry of habitat patches or their historical proximity. Over time, as habitats within this historical configuration, became smaller and lesser in number, one would expect that the past regional pool of species starts to differentiate due to a combination of dispersal limitations and habitat changes taking place. However, our study suggests a remnant of the past species similarities remained in the contemporary pairwise beta diversities (Chase, 2003). This remnant similarity can be indicative of an extinction debt that will be paid off in the future as the species compositions further diverge, due to a decrease in immigration events between habitats (Zelnik et al., 2018). Conversely, for odonate species, a 70‐year time‐lagged response to past landscape change may seem too long for a species with a generation time of a few years. Nevertheless, Rocha‐Ortega et al. (2019), tried to identify time‐lagged responses to land degradation in odonate species over a 30‐year time period and finally concluded that such time lags probably took place over longer periods of time. In addition to generation times influencing the period over which a response is lagged, habitats that were historically part of a dense network of patches may show similarities in physical and chemical properties and may thus also support a similar species pool over longer periods of time. Network structures additionally can have a certain delayed disintegration due to redundancies in network connectivity, for example, connectivity through stepping stones if direct movement is inhibited (Economo & Keitt, 2010). Therefore, extinction debts might occur over a larger time span in species regional diversity than in local diversity. This, however, needs further investigation.

## 5 CONCLUSION

At the interface of metacommunity dynamics, landscape history and network theory, this study identifies metacommunities based on their historical geographical proximities. As new layers of spatial data are consistently being created, not just of the current but also of landscape history, it is imperative to include more of history into our analyses to enable better informed diversity conservation in dynamic landscapes. Our proposed method of estimating metacommunity spatial extents allows for a generalisable method that can be used to plan regional ecological infrastructure networks to conserve metacommunity spatial structure. Additionally, our study shows the importance of including historical data for this purpose since it shows a higher accordance to species diversity than the current habitat information. To conclude, metacommunity analysis using habitat network history can be a useful knowledge base for estimating metacommunity spatial extents at a regional scale. This can also help identify regional landscape units within which collaborations between multiple stakeholders can be fostered to holistically manage the landscape for biodiversity conservation. Our approach and future developments of it can be useful in conservation planning to move beyond static landscapes and integrate the dynamics of long‐term landscape change into conservation actions.

## AUTHOR CONTRIBUTIONS

**Nivedita Varma Harisena:** Conceptualization (equal); data curation (lead); formal analysis (lead); investigation (lead); methodology (equal); project administration (equal); software (lead); validation (lead); visualization (lead); writing – original draft (lead); writing – review and editing (equal). **Adrienne Grêt‐Regamey:** Conceptualization (supporting); project administration (equal); supervision (equal); writing – review and editing (equal). **Maarten J. Van Strien:** Conceptualization (equal); funding acquisition (lead); methodology (equal); project administration (lead); resources (lead); supervision (lead); writing – review and editing (equal).

## FUNDING INFORMATION

This research was conducted under the EMPHASES project (https://plus.ethz.ch/research/forschungsprojekte/SNF_EMPHASES.html) funded by the Schweizerischer Nationalfonds (SNF) with grant no. 200021_192018.

## CONFLICT OF INTEREST STATEMENT

The authors declare that they have no known competing financial interests or personal relationships that could have appeared to influence the work reported in this paper.

## Supporting information


Appendix S1.

## Data Availability

The data on odonate species richness and time series of landcover information is available from InfoFauna and the Institute of Cartography of ETH Zurich, respectively. Restrictions apply to the availability of these data, which were used under licence for this study. Code used to analyse the data will be available at https://github.com/NVHarisena1/Metacommunity_delineate_classify.git.
