# Supplementary material for: Identification of metacommunities in bioregions with historical habitat networks
Source: Ecol Evol. 2024 Aug 8;14(8):e70076. doi: 10.1002/ece3.70076 (PMC11309940; doi:10.1002/ece3.70076)
Supplement: Supplementary file 1 — Appendix S1. [file ECE3-14-e70076-s001.docx]

## APPENDIX

**APPENDIX A: Patch number, area and fragmentation index for wetlands within the Swiss Plateau for 1850,1900,1950,2010** (Rivas et al., 2022)

|  | Number of patches | Total area of patches (sqm) | Mean area per patch (sqm) | Patch Fragmentation Index * |
| --- | --- | --- | --- | --- |
| 2010 | 3509 | 50420000 | 14368.76603 | 0.611808031 |
| 1950 | 5916 | 198919375 | 33623.96467 | 0.384350048 |
| 1900 | 8563 | 467116250 | 54550.53719 | 0.264245248 |
| 1850 | 9346 | 787146875 | 84222.86272 | 0.109442387 |

*Higher value implies more fragmentation

**APPENDIX B: Historical networks with different dispersal thresholds and impact on beta diversity classification**

B1. 250m dispersal threshold

**
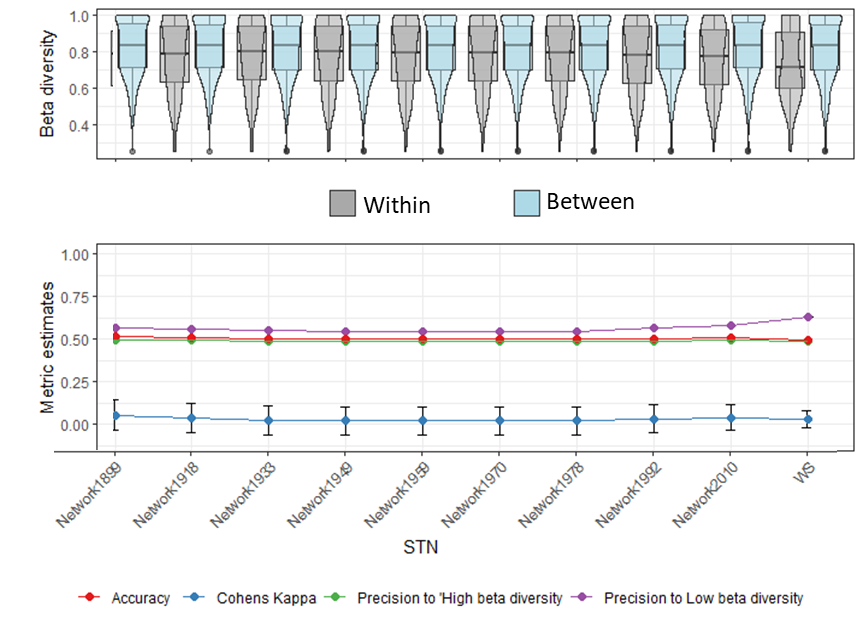
**

B2. 750m dispersal threshold

**
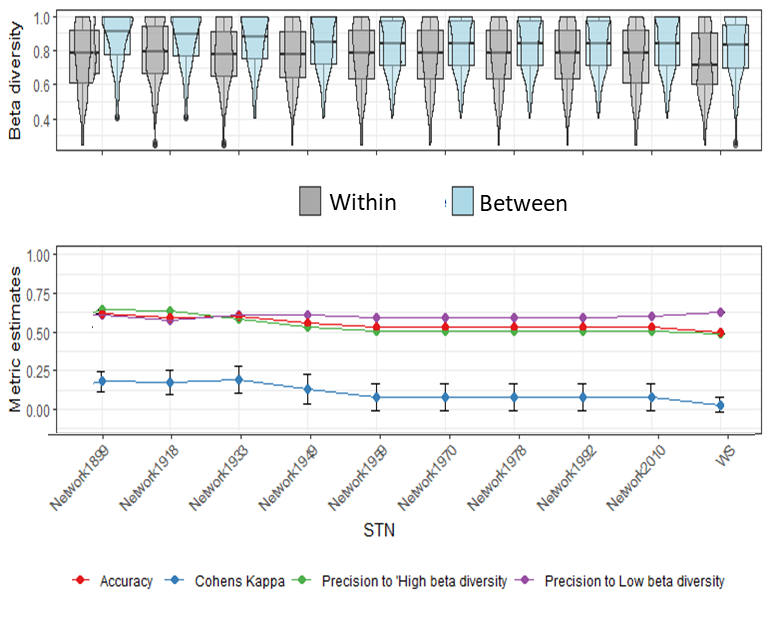
**

B3. 1000m dispersal threshold

**
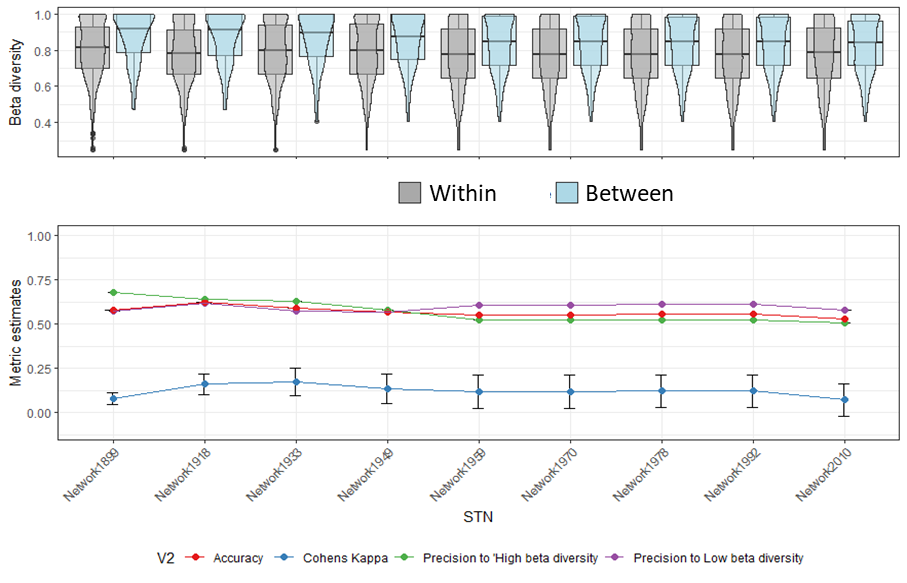
**

B4. 2000m dispersal threshold

**
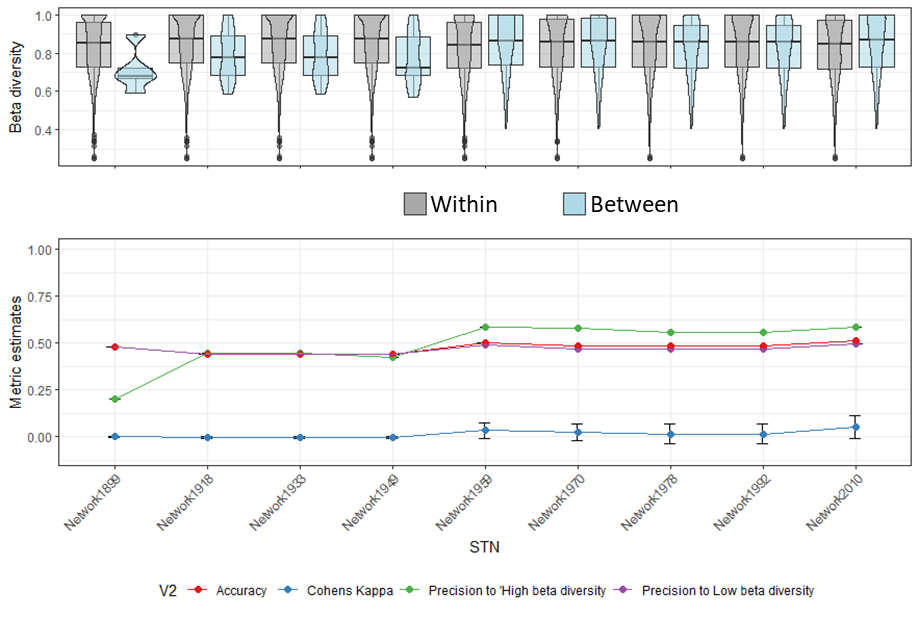
**

**APPENDIX C: T-test p values for “between” and “within” groups for different metacommunity definitions at 500 m dispersal limit**

*grey highlight significant at 99%; light grey significant at 90%
